# Automated error localisation and correction techniques for deep-learning-based segmentation of 3D MRI sequences based on feature-derived-region aggregation

**DOI:** 10.1038/s41598-026-57383-8

**Published:** 2026-06-12

**Authors:** Adrian C. Ruckli, Valentin Roesler, Hanspeter Hess, Malin K. Meier, Florian Schmaranzer, Nicolas Gerber, Simon D. Steppacher, Kate Gerber

**Affiliations:** 1 Department of Orthopaedic Surgery and Traumatology, Inselspital, University of Bern, Bern, Switzerland; 2 Department of Diagnostic-, Interventional- and Pediatric Radiology, Inselspital, University of Bern, Bern, Switzerland; 3 Department of Radiology, Balgrist University Hospital, University of Zurich, Zurich, Switzerland

**Keywords:** Deep Learning, Automatic Segmentation, Uncertainty Estimation, Error Localisation, Clinical Metric, 3D Hip MRI, Computational biology and bioinformatics, Engineering, Health care, Mathematics and computing, Medical research

## Abstract

Automatic segmentation using convolutional neural networks (CNNs) has become a key tool in musculoskeletal imaging, offering substantial reductions in processing time. However, concerns about reliability often necessitate manual inspection and correction. We present a method that leverages network-derived uncertainty to automatically identify and localise segmentation errors, reducing the need for exhaustive manual review. A 3D nnU-Net was trained on delayed gadolinium-enhanced MRI of hip cartilage. Voxel-wise uncertainty scores, computed from the SoftMax outputs of ensembled sub-networks, were aggregated over feature-based supervoxels. Each region was then evaluated for its potential impact on clinically relevant metrics, generating sensitivity scores. A logistic model combined these with uncertainty data to assign risk scores, guiding attention to areas most likely to affect clinical metrics during the initial correction steps. Using these risk scores, guided supervoxel correction of just 50 supervoxels reduced the mean absolute relative error by 2.1-fold. Guided manual correction within these regions achieved a 3.5-fold reduction, an approximate 62% supervoxel correction efficiency. Correcting the top 10 regions yielded up to 88% efficiency. This approach serves as a proof-of-concept for targeted correction in hip MRI, enhancing the clinical utility of CNN-based segmentation by demonstrating that 3D feature-derived uncertainty aggregation has the potential to reduce correction burden compared to traditional 2D methods.

## Introduction

Recently, the automatic semantic segmentation of medical images using convolutional neural networks (CNNs) has become more prevalent. In this approach, CNNs trained with manual segmentation data are subsequently used to infer the segmentation of images not used in the training of the model, eliminating the need for time-consuming manual segmentation of data. Using network architectures designed for medical image segmentation, such as the U-Net^1^, automatic multi-structure segmentation with average accuracies equivalent to manual segmentation has been enabled for a range of anatomical regions^2^. Once a model has been trained, segmentation can be performed in a matter of seconds, facilitating the incorporation of more quantitative three-dimensional (3D) analysis into the clinical routine.

The demand for highly accurate automatic segmentation spans a wide array of complex medical imaging tasks, ranging from prostate cancer radiomics and the detailed segmentation of cerebrovascular networks, to PET-based 3D tumor volume reconstruction and 3D cellular structures^3–6^. Across all such diverse applications, ensuring the reliability of the extracted quantitative data is critical. The robustness of these deep learning-based segmentation algorithms, however, remains inferior compared to expert manual segmentation in practice, and segmentation networks might produce inadequate segmentations when the input image data is dissimilar from the data used to train the model. The use of MRI scanners from various manufacturers and different imaging protocols employed by different clinics can result in variations in image intensities and resolutions that might diverge from the input data used for training. The inherent heterogeneity of the imaged anatomy can also result in segmentation failure. Due to this subpar robustness, there are concerns relating to the integration of these techniques into clinical routine, as users would currently be required to review each segmentation, slice by slice, to ensure adequate segmentation quality. If segmentation failures are identified, users must manually segment the affected regions. This time-consuming process, which requires specialised skills and software, detracts from the benefits gained from these automatic segmentation networks^2,7–9^.

While ongoing research is dedicated to improving the robustness of these automatic segmentation models, another research trend focuses on the detection of segmentation failures by analysing network-generated uncertainties. Recently, algorithms have been proposed to detect the segmentation failures of a given structure^10–12^. These algorithms, which have shown promising results, exploit network-generated uncertainties to predict the segmentation quality of each target structure. We recently introduced such a method, based on the nnU-Net framework, that detected segmentation failure with a sensitivity of 1.0 and specificity of 0.94^10^. This structure-wise failure detection is helpful for an expert to flag affected segmentations. However, these methods do not localise the error within the segmentation and are therefore unable to assess the clinical significance of the error. Segmentation failure can be regional and might not be pertinent to the quantification of the relevant clinical metric. Furthermore, a flagged segmentation still requires the time-consuming inspection and manual correction of each slice containing the structure.

In segmentation, where a class label is assigned to each voxel, the network-generated uncertainty typically reflects the confidence level of the predicted class label. However, these voxel-associated confidences may not accurately represent the misclassification probability of a given voxel. Voxel-associated uncertainties may be leveraged to localise segmentation errors if the confidences are well calibrated (i.e., represent the probability of misclassification). For brain tumour segmentation, confidence calibration of different uncertainty metrics has been investigated by Jungo et al.^9^. While they reported that calibration for voxel-wise predictions was unreliable, they did find that segmentation failure detection on the patient level is possible by aggregating voxel-wise uncertainty estimates. The best results were achieved by aggregating features extracted from the original image. They hypothesised that aggregating voxel-wise uncertainties could be an important step for localising erroneous regions within a segmented structure. Several other proposed approaches also leverage the aggregation of uncertainties. In these approaches, the uncertainties of all voxels contained in a predefined region are used to calculate a scalar confidence score for the respective region. By aggregating voxel-wise uncertainties, the influence of deficiently calibrated voxels can be reduced^11–15^. Another approach involving aggregation has been proposed by Sander et al.^7^. They assessed the voxel-wise uncertainty in two ways: using the entropy from the SoftMax output of the automatic segmentation model, and using a measurement of the standard deviation derived from Monte Carlo dropout (MC-dropout)^16^. The voxel-wise uncertainty was then aggregated over a rectangular sliding window. In conjunction with the original image, the voxel-wise uncertainty map was utilised as input to an auxiliary CNN, which assigns an uncertainty score to the window region. They integrated this error localisation into a guided correction approach and claimed that the correction process could be expedited with this technique. However, a two-dimensional (2D) rectangular aggregation window disregards the context of the original image and does not make use of prior knowledge of image features. This approach is also limited to MRI sequences with a high slice thickness, high image contrast, and lower slice numbers than high-resolution 3D mapping sequences. Employing a feature-derived aggregation region in three dimensions could potentially partition uncertainties into more contextual regions, likely enhancing the utility of the identified regions for semi-automatic correction techniques.

A semi-automatic correction technique in which a user can indicate segmentation failures with mouse clicks and scribbles was proposed by Wang et al.^17^. From this input, geodesic distance maps were generated and used as input for a CNN together with the original image and segmentation. Their technique achieved 3D corrections from user interaction on 2D MRI slices. They demonstrated that their method could expedite the correction process and achieve better results than unaided segmentation models. Xie et al.^18^ built on this approach, using network-generated uncertainties instead of user interactions to obtain an error map. They then applied an auxiliary model for the re-segmentation of regions likely containing a segmentation error. They showed that this approach could increase the accuracy of the segmentation. However, network-generated uncertainties can only predict which regions are likely to contain a segmentation error. Some user interaction would, therefore, likely be beneficial in confirming which regions are actually erroneously segmented.

Previously reported correction techniques have been designed for use on the entire structure and do not consider the impact of the correction on the final application. In clinical applications, segmentations are often used for anatomy analyses that are limited to certain regions of the anatomical structure or for treatment planning that depends on accurate anatomy segmentations in specific anatomical regions only. Thus, the importance of segmentation accuracy is not always uniform over the whole structure. Undertaking corrections of the entire structure may thus be an unnecessary effort that has the potential to divert attention from more critical regions. We propose that incorporating information pertaining to the impact of the regional segmentation accuracy on the final metric for which the segmentation is used could help to optimise and guide correction. The impact on the final metric can be evaluated on the same feature-derived regions needed for the uncertainty aggregation. Knowing the impact of regional segmentation on the final metric is also important for determining when a region is likely to be sufficiently accurate for the specific application.

To overcome the described limitations of current localisation and correction approaches and to limit required correction to clinically important regions, we propose to combine network uncertainty aggregation over 3D feature-derived regions with an estimation of the impact on the clinical end-metrics. Supervoxels^19^ are proposed to be used as they are a 3D structure, feature-derived from the MRI, and simple to understand and interpretable for the end user. Such regions could be used as alternatives to single voxels^20^ to guide users’ attention during correction or to guide semi-automatic correction mechanisms for the correction of a complete region.

In this work, we investigated the efficacy of our proposed approach as a foundational proof-of-concept for potentially reducing the correction burden in musculoskeletal imaging, specifically deep learning-based segmentation of the hip cartilage and labrum from MRI. For the treatment planning of femoral acetabular impingement (FAI)^21^, we recently presented methods for the automatic analysis of cartilage volume, surface area, and thickness from MRI^22^ using deep learning based segmentation. While the trained nnU-Net^23^ enabled average segmentation accuracies comparable to an expert inter-rater agreement^24^, the distribution of errors across patients was not uniform. In particular, some segmentations failed significantly, especially for atypical anatomies or poor image quality. These failures are masked by the mean accuracy metric, which can give a misleading sense of reliability. As a result, without appropriate mechanisms for error detection and correction, such a system could produce clinically unacceptable results in individual cases, despite appearing to perform well on average.

We hypothesised that combining voxel-wise segmentation uncertainties, aggregated over feature-derived regions, with an estimate of the clinical metric variation, could be used to prioritise, or guide, the correction of localised regional segmentation errors. This approach aims to reduce the overall correction effort while effectively reducing errors in the clinical metrics.

## Methods

**Fig. 1:**
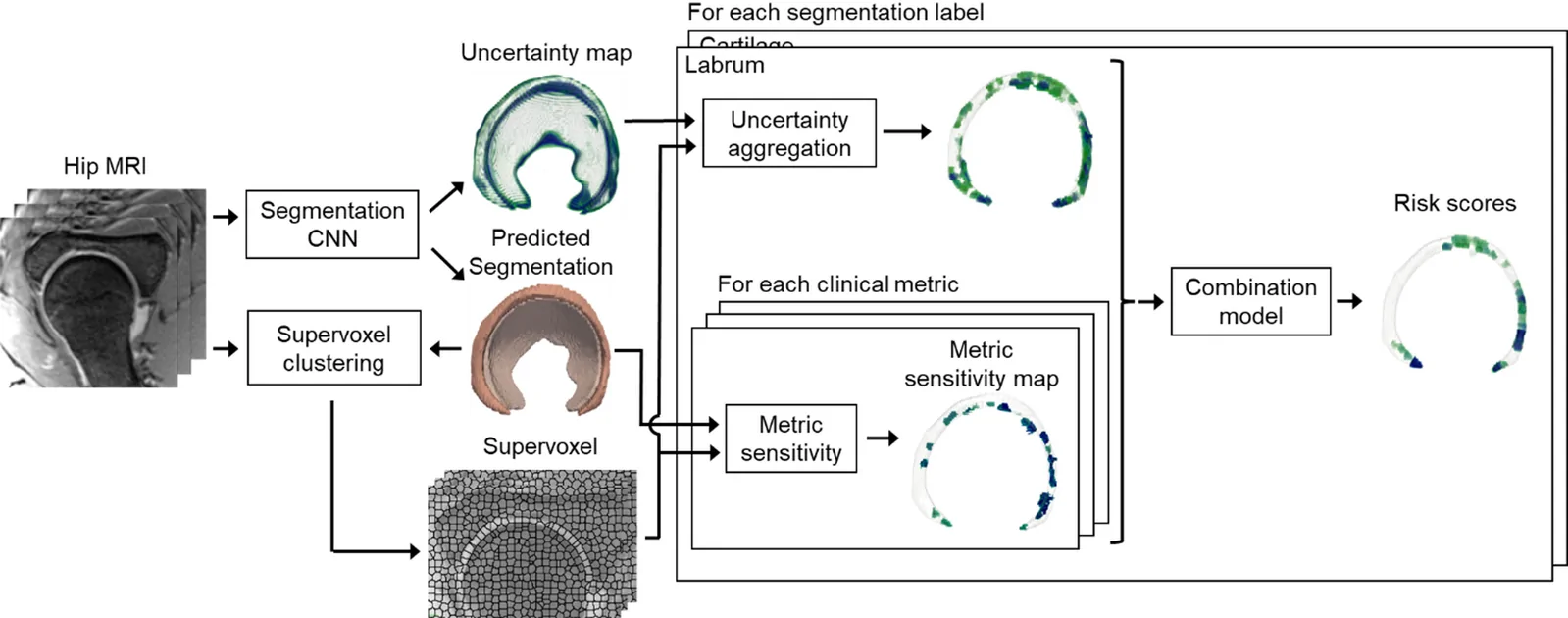
Overview of the proposed segmentation failure detection method. Left: Automatic segmentation of cartilage and labrum from hip MRI combined with assessment of segmentation uncertainties. The MRI and the predicted segmentation are used to calculate supervoxels. Right: The voxel-wise uncertainty is aggregated over the supervoxel. The sensitivity of each region defined by the supervoxel on the clinical metric is estimated, and a map is generated. The aggregated uncertainty map and the sensitivity maps are combined to predict segmentation failures. Each metric has its sensitivity map, and each label has its segmentation failure prediction. Blue indicates a higher uncertainty or sensitivity, and green a lower one.

In this study, we validated the use of risk scores, combining segmentation uncertainty with clinical metric sensitivity estimates, to spatially prioritise segmentation error correction. Epistemic uncertainties^25^, generated by a CNN during segmentation of the hip cartilage and labrum from CT, were aggregated over feature-derived regions and combined with sensitivity maps defining the effect of regional segmentation error on anatomical morphological parameters (Fig. 1).

### Data

With the approval of the local ethical commission (Kantonale Ethikkommission Bern, Switzerland, KEK 2022−00618) and a waiver for informed consent, a dataset comprising 100 MRIs of the femoral head, previously described by Meier et al.^24^, was used for this study. Inclusion criteria were a symptomatic hip deformity, patients aged > 18 years, and an MRI scan according to the institutional routine protocol. The MRI data were acquired with a 3T unit (Magnetom Skyra, Siemens Healthineers) and included an axial-oblique 3D T1 MP2RAGE^26^ sequence with a voxel spacing of 1.0 × 0.5 × 0.5 mm from which the labrum and cartilage were manually segmented by clinical experts. The image volume was automatically cropped to 80 × 200 × 200 voxels around the femoral head centre, defined by fitting a sphere to the manual cartilage segmentation. The dataset was randomly split into training, calibration, and test sets according to a 60%, 15%, and 25% split, respectively. This study was performed in accordance with all relevant guidelines and regulations, including the Declaration of Helsinki.

## Segmentation and uncertainty assessment

### Automatic anatomy segmentation

For automatic segmentation of the 3D hip cartilage and labrum structures from the MRI^24^, a 3D U-Net^1^ was trained and evaluated using the self-configuring nnU-Net framework from Isensee et al.^23^ with the MRI and corresponding manual segmentations of the training data set (*N* = 60). The deep learning network architecture includes encoder and decoder paths, connected via skip connections, as well as a bottleneck at the lowest layer. The base building block consists of a convolution layer followed by an instance normalisation layer and a leaky rectified linear unit (negative slope, 0.01) as an activation function. Two consecutive building blocks were used for each resolution step. The first layer in the encoder and decoder paths operated exclusively on the axial planes, adopting a pseudo-2D configuration to create or extract from isotropic image features efficiently. In the nnU-Net, upsampling is implemented as transposed convolution. The initial feature map consisted of 32 channels and was doubled with each downsampling operation (strided convolution), limited to a maximum of 320 channels. The patch size was set to 64 × 192 × 160 voxels. Segmentation masks were generated with a 1 × 1 × 1 convolution and a SoftMax layer followed by an ArgMax operation for background, cartilage, or labrum tissue. The network was trained with deep supervision; additional auxiliary losses were added in the decoder to all but the two lowest resolutions, allowing the gradients to be injected deeper into the network and facilitating the training of all layers in the network^24^. The network was trained in five folds for 60 epochs with initial random weights. One epoch is defined as an iteration over 250 mini-batches (consisting of two patches). Stochastic gradient descent with Nesterov momentum ($\:\mu\:=0.99$) and an initial learning rate of 0.01 was used. The learning rate is decayed throughout the training following the formula $\:{\left(1-\mathrm{epoch}/{\mathrm{epoch}}_{\mathrm{max}}\right)}^{0.9}$. The loss function is the sum of cross-entropy and Dice loss. The standard network configuration for the application of the trained model to unseen data was defined as the ensemble of the 5 networks trained during the 5-fold cross-validation.

### Segmentation failure and simulated correction

**Fig. 2:**
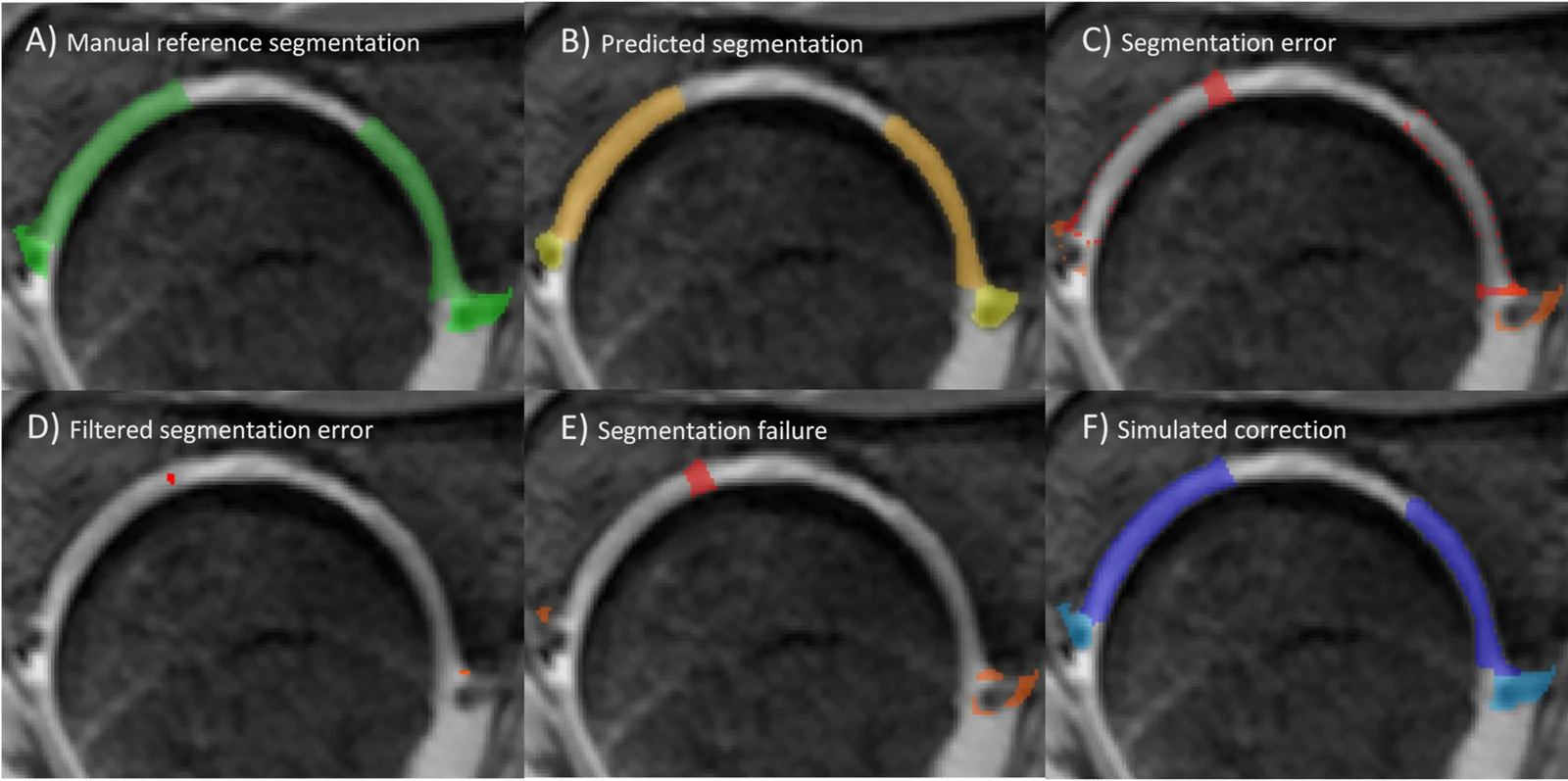
Axial oblique view of cartilage and labrum segmentation, showing segmentation errors and segmentation failures. (A) Manual reference segmentation; (B) Predicted segmentation; (C) Segmentation error – difference between A and B; (D) Filtered segmentation error after applying a distance filter and one erosion step; (E) Segmentation failure – regions obtained by dilating D within error mask C; (F) Simulated correction: corrected version of B using E as the correction mask.

The trained network was used to automatically segment the cartilage and labrum from all MRI datasets in the calibration and test datasets. Segmentation errors (Fig. 2C) of automatically generated segmentations were calculated by comparing the predicted segmentation (Fig. 2B) with the manual segmentation (Fig. 2A). Furthermore, based on knowledge based filtering maps of relevant segmentation failures were generated as proposed by Sander et al.^7^, by removing errors within the ranges of inter-observer variabilities or errors which would typically not be corrected by a user, such as individual pixels at the segmentation surface. For each anatomical structure in each MRI, an error mask (Fig. 2C) and a 3D distance transform map were computed based on the distance of the predicted anatomical structure. Small segmentation errors closer than 0.5 mm (smaller than the through-plane and larger than the in-plane resolution) presumably within the range of the inter-observer variability^7,24^, were filtered out, followed by one iteration of erosion (Fig. 2D) to remove very thin remaining parts. The remaining errors outside of the tolerance threshold were used as seeds (Fig. 2D) for a binary dilation within the original error mask (Fig. 2C) until no further changes. This ensured that the regions of segmentation failures (Fig. 2E) were complete and remained connected to the boundary of the reference structure. To simulate corrected segmentations (Fig. 2F) made by an expert reader, the segmentation failure maps were used to correct the automatic segmentation predictions by copying the content from the manual segmentation to the automatic segmentation for voxels contained within the failure map. The resulting segmentations were used as reference segmentations.

### Clinical metric calculation

**Fig. 3:**
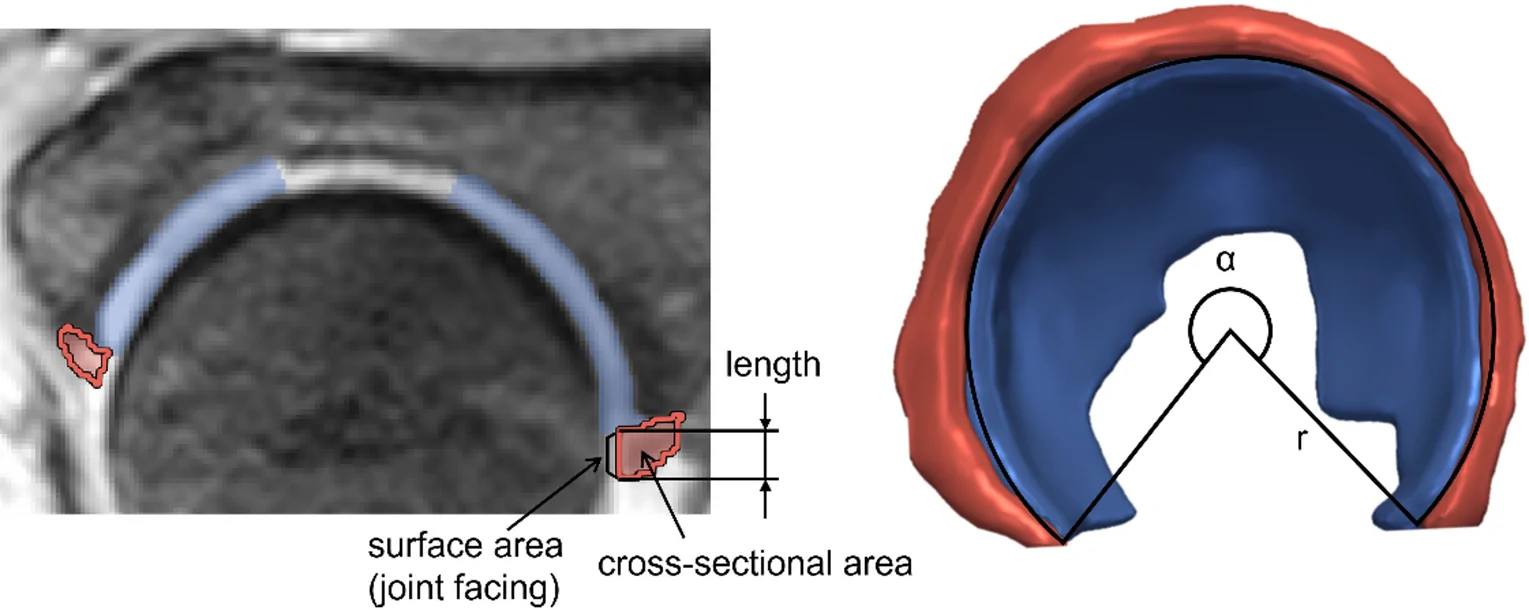
Chondrolabral complex geometric definitions for metric computation. Left: MRI slice with segmented cartilage (blue) and labrum (red). Right: 3D reconstructions. A best-fit sphere to the femoral head provides the radius (* r*); the labral clockface coverage is the angle (α). The labral arc length is $\:L=\alpha\:r$, used to compute the average labral cross-section $\:{V}_{labrum}/L$ and average labral length $\:{A}_{labrum}/L$.

Based on the segmented 3D anatomical models, the cartilage volume, surface area, and thickness, and the labrum volume and surface area, were calculated according to our previously published methods^22,24,27^ for each case in the calibration and test datasets. The average cross-section and average length of the labrum were additionally calculated by dividing the labrum volume and the surface area by the product of the angular coverage (α) on the clockface and the femoral head radius (\:r), both estimated from a fitted sphere (Fig. 3).

### Voxel-wise uncertainty analysis

We previously showed that the uncertainty of the nnU-Net’s default deterministic subnetworks (*N* = 5) created during the cross-fold validation could be used to assess structure-wise uncertainty without significant additional computational cost^10^. In this work, we augmented the approach to allow for voxel-wise uncertainty analysis using the mean SoftMax map prediction from the ensemble. The maximum value of the SoftMax per voxel was used to define the confidence of the label prediction.

To assess the calibration accuracy of the different confidence values against the segmentation errors and segmentation failures, reliability diagrams^28,29^ depicting the associated expected calibration error (ECE)^30^, the average calibration error (ACE)^31^, and the maximum expected calibration error (MCE)^30^ were generated. Jungo et al.^9^ showed that a good, calibrated network on the dataset level can still experience large calibration error for individual images on the patient level; thus, we calculated the ECE, ACE, and MCE per patient and calculated the average over the dataset. For each prediction, we also measured the Dice coefficient to assess the accuracy of the prediction. To address a general over- or under-confidence, we used temperature scaling to improve the calibration accuracy^29^. The temperature (*T*) was optimised with respect to the binary cross-entropy loss over all voxels of the calibration dataset.

We evaluated the segmentation accuracy and calibration accuracy from the standard ensemble configuration against a single network with activated test time dropout (TTD). The dropout layers were located after each convolutional layer. TTD is widely used as a Bayesian approximation with a single network to estimate epidemic uncertainties^32,11,33,34^.

Network settings with TTD allowed multiple stochastic predictions (*N* = 1 to 5) with different dropout probabilities (*p* = 0.05, 0.10, 0.15, 0.20). The stochastic predictions were averaged to generate a final SoftMax output. Two different location settings for the dropout layers were used: (1) only in the encoder path and (2) in both the encoder and decoder paths. Predictions with TTD were repeated 3 times, and the average of the runs was calculated.

We tested the standard ensemble and the stochastic network results with enabled TTD, with and without the built-in test time augmentation (TTA). TTA, which mirrors the image patches in all axes and averages the predictions for each configuration, generates eight times more predictions with 3D patches and increases computation time by approximately a factor of 8.

### Feature-derived regions

Supervoxels were calculated for each MRI scan using the simple linear iterative clustering (SLIC) algorithm^19,35^, to obtain feature-derived agglomeration regions. The algorithm first partitions the input image volume into a grid of cuboids of a specified dimension. The centres of these clusters are then iteratively shifted by applying k-means until they optimally partition the volume into voxel groups of similar contrast and spatial proximity. The effects of two parameters, grid size and proximity weight, on the generated supervoxels were investigated on the calibration dataset. The grid size determines the dimensions of the initialisation cuboids, specified as a vector of depth, width, and height in voxel coordinates. Higher values result in fewer supervoxels with bigger volumes. The proximity weight determines the influence of spatial proximity during the k-means clustering. Therefore, when the proximity weight is high, voxels in close proximity might be clustered together even when their intensities are not very similar. The proximity weight parameter further influences the shape of the resulting supervoxels. Higher values may result in more compact and regular shapes of the generated supervoxels. For a semi-automatic correction approach, the borders of the supervoxels should be aligned with the automatically predicted segmentation. To achieve this, we used the segmentation image as an additional input channel for the SLIC algorithm.

### Aggregating uncertainties

For each pair of MRI and segmentation images of the calibration dataset, nine different maps of aggregated regions were created by applying SLIC with different combinations of grid size and proximity weight parameters. Grid sizes were chosen to yield cubic initialisation regions. Considering the 1:2 ratio of the through- and in-plane resolutions, grid sizes of 2 × 4 × 4, 3 × 6 × 6, and 4 × 8 × 8 were chosen, and proximity weights of 10, 100, and 1000 were applied. The uncertainty of each aggregated region was defined as the average of the individual voxel uncertainty values (normalised entropy from the SoftMax) contained within the supervoxel.

### Segmentation failure localisation

To assess the sensitivity of the clinical metrics to segmentation failures, we automatically generated a spatial sensitivity map, * m(s,l)*, for each label (* l*) based on the supervoxel (* s*), for each corresponding metric. The contribution of each supervoxel to the metric was assessed by sequentially including and excluding it from the segmentation result. The relative sensitivity was then calculated as the difference in the metric value from the modified segmentation, divided by the initial metric value from the original automatic segmentation. To reduce computational costs, only supervoxels in contact with the initially predicted structure were considered.

To assess the spatial risk score, we combined the aggregated uncertainty map and the metric sensitivity map in a supervoxel-wise logistic model, $\:{p}_{logistic}(s,l)$ according to Eqs. 1,

1 $$\:{p}_{logistic}(s,l)=\:\frac{1}{1+{e}^{-{\beta\:}_{0}-{\beta\:}_{1}\cdot\:{m}_{1}(s,l)-{\beta\:}_{2}\cdot\:{m}_{2}(s,l)-\dots\:\:-{\beta\:}_{k}\cdot\:{m}_{k}(s,l)}}$$

where $\:{\beta\:}_{0}$ is the bias term, $\:{\beta\:}_{1}\:$is the coefficient for $\:{m}_{1}(s,\:l)$, which equals to 1 if the supervoxel belongs to a different segmented structure, and 0 otherwise, $\:{\beta\:}_{2}\:$is the coefficient for the aggregated uncertainties ($\:{m}_{2}(s,\:l)$). $\:{\beta\:}_{3}\:$to $\:{\beta\:}_{k}$ are the coefficients for each metric sensitivity map. $\:{m}_{3}(s,\:l)$ to $\:{m}_{k}(s,\:l)$ are the spatial metric sensitivity maps based on supervoxel, respectively. For the minimising process, $\:{\beta\:}_{0}$ and $\:{\beta\:}_{1}$ were limited between − 10 and 10. $\:{\beta\:}_{3}$ to $\:{\beta\:}_{k}$ were limited to be ≥ 0.

**Fig. 4:**
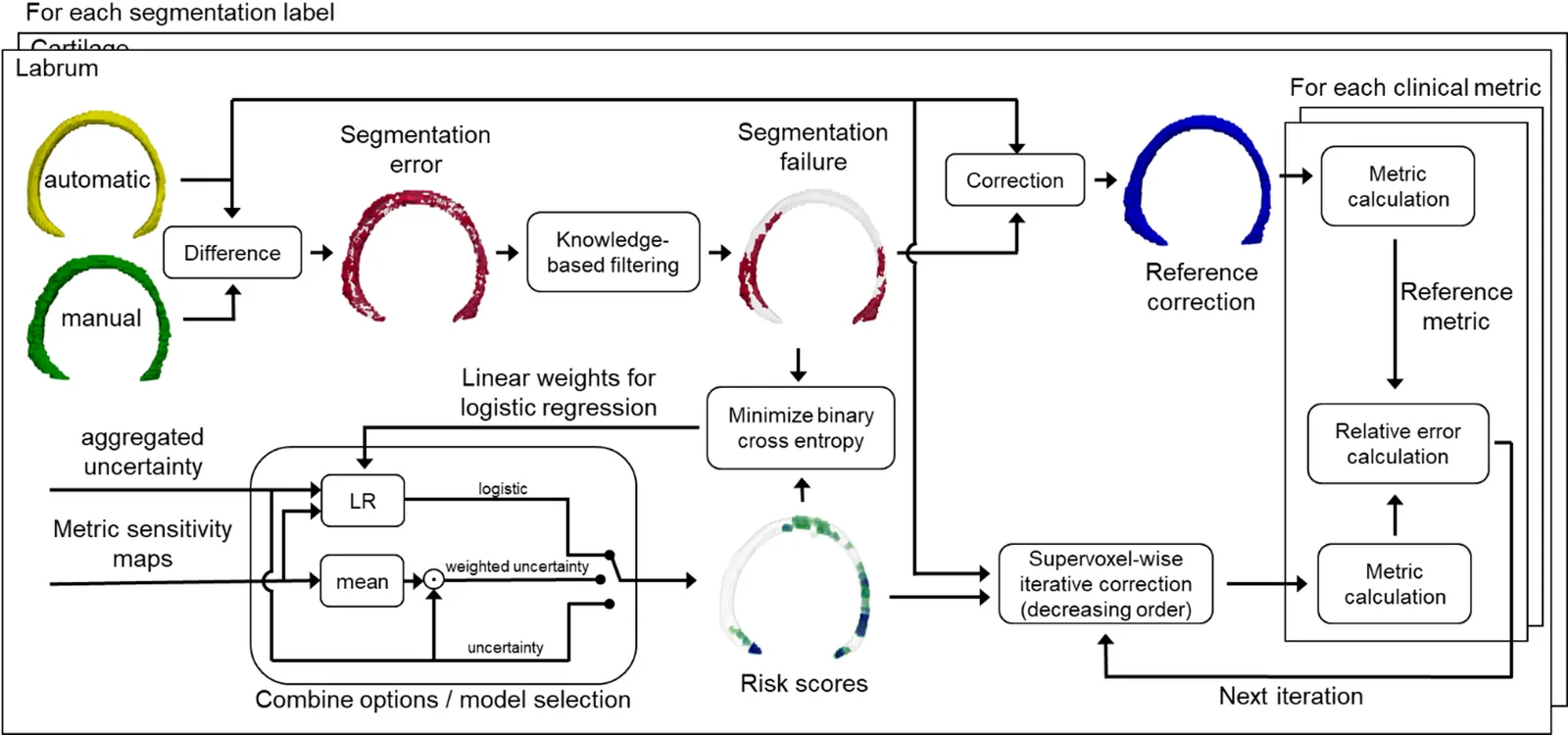
A detailed overview of the process for predicting segmentation failures and supervoxel-wise corrections is presented. Top path: The segmentation error is determined by comparing the manual segmentation with the automatic segmentation from the convolutional neural network. Using prior knowledge, only the segmentation failures are retained from the identified errors. These segmentation failures are then used to correct the initial automatic segmentation, which serves as a reference correction for further analysis of clinical metric corrections. Bottom path: The aggregated uncertainty maps and metric sensitivity maps were combined using 3 different approaches: the logistic model, the pure uncertainty map, and the metric-weighted uncertainty map. The weights for the logistic model are calculated by minimising the binary cross-entropy between the calculated segmentation failure maps and the predicted segmentation failure maps over the calibration set. Based on these predicted segmentation failure maps, iterative corrections at the supervoxel level were applied and evaluated at the metric level.

In addition, we added a map defining if a supervoxel belongs to another segmented structure to help define weights for errors of target structures versus those of the background and the structure of interest. The regression coefficients were defined by minimising the binary cross-entropy loss between the results of the logistic model and segmentation failures using the L-BFGS-B algorithm (Fig. 4). Because our calibration data set was generated from a random split of the complete dataset, and our interest was in the localisation of errors, primarily in poorly segmented cases, we weighted the images for the minimisation process based on the number of voxels in the segmentation failure map. The image with the largest number of wrongly segmented voxels was assigned a weight of 1.0, and an image without any failure was assigned a weight of 0.1. The other weights were interpolated linearly.

The approach thus assigns a predicted risk score with scalar values ≥ 0 to each supervoxel per segmented structure.

### Segmentation failure correction

To evaluate how our proposed segmentation failure localisation method could guide segmentation corrections to increase its efficiency, we simulated a correction process where the segmentation of the supervoxels with the highest risk scores is proposed to be corrected first. If these predicted risk scores are accurate, it is expected that corrections lead to a rapid reduction in the clinical metric’s error initially, followed by diminishing reduction with subsequent corrections.

On the calibration dataset, we compared the performance of three different methods for segmentation failure localisation, the logistic model, the aggregated uncertainty map and a weighted uncertainty-based approach, where the aggregated uncertainty map was weighted by the mean of the metric sensitivity maps (Fig. 4).

Two different semi-automatic correction methods were simulated to evaluate the performance of the error localisation methods. The first method simulates an approach in which the supervoxels are used only for failure localisation, and the correction of each voxel within the supervoxel is performed manually. The voxels contained by the treated supervoxel in the reference correction are copied to the predicted segmentation, effectively removing any error from this region. This method is called *guided manual correction*. The second method simulates a *guided supervoxel correction* approach in which all voxels in the supervoxel are corrected to the value of the label which is assigned to the most voxels within the supervoxel region in the reference correction.

For each iterative correction step, we evaluated the sensitivity of the method in detecting the segmentation failures and evaluating the clinical metrics (Fig. 4, right). The mean absolute relative error (MARE) of each clinical metric calculated from the automatic segmentations and reference segmentations, from all patients in the respective data subset, was calculated.

The calibration set was used to evaluate the network settings for predictive uncertainties, to optimise the logistic model, and to evaluate the methods for predicting segmentation failures. The test set was used to evaluate the selected models and settings on unseen data. Given the possibility for structure-wise error calculation for identification of segmentation with high error prior to error localisation^10^, we divided the test set into two evaluation groups: those with above-average and those with below-average segmentation accuracy, based on the Dice coefficient.

## Results

Compared to the reference segmentations, the trained model achieved an average Dice score of 97.6% for cartilage and 93.9% for labrum on the test dataset. Prior to any correction. MARE were 3.2% for cartilage volume, 2.4% for surface area, and 0.9% for thickness. For labrum, the MARE values were 7.5% for volume, 5.2% for surface area, 6.6% for cross-section, and 4.1% for length.

### Assessment of predictive uncertainties

**Fig. 5:**
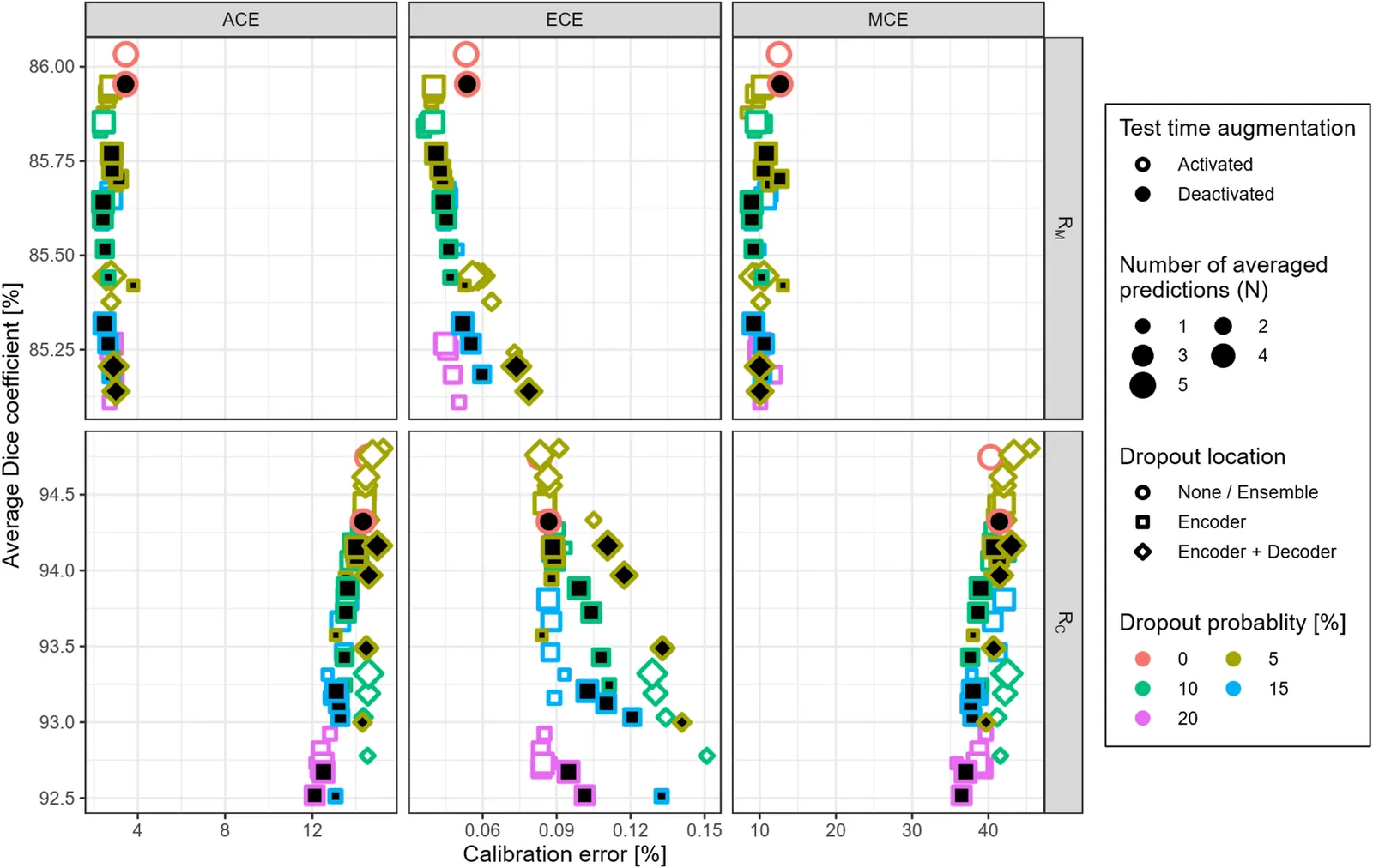
Calibration error results on the calibration set against the manual reference (R_M_) (including the manual segmentation and the segmentation errors), and the correction reference (R_C_) (including the corrected segmentation and segmentation failure). For R_C_ and R_M,_ only values with a mean Dice of ≥ 92% and ≥ 84% are shown, respectively. The mean Dice from the cartilage and the labrum was averaged over the calibration set. Stochastic results with test time dropout (TTD) were repeated 3 times and averaged. Average calibration error (ACE), expected calibration error (ECE), and maximum expected calibration error (MCE) were calculated per patient and averaged over the calibration set. TTD was evaluated using different numbers (N) of averaged SoftMax results, different dropout probabilities (p), and dropout locations. Test time augmentation (TTA) was applied to the TTD results and the standard nnU-Net ensemble results. The upper-left corner of the plot indicates the optimal region.

Overall, the standard ensemble of the 5-fold of the nnU-Net performed better than other tested configurations, providing the highest segmentation accuracies for the cartilage and labrum compared to the manual segmentation on the calibration dataset. Maintaining high segmentation accuracies, MC-dropout with activated TTD, TTA, and low dropout chances of 5% reduced the calibration error of confidence estimates for segmentation errors (relative to manual segmentations) compared to the standard ensemble, but this improvement did not extend to segmentation failures (relative to simulated corrected reference segmentations). A reduction in the calibration error for segmentation failures was linked to a decrease in segmentation accuracy. The detailed results are provided in Fig. 5. Based on its good performance, deterministic results, and fast segmentation time, the standard ensemble without TTA was used for the remainder of the study.

**Fig. 6:**
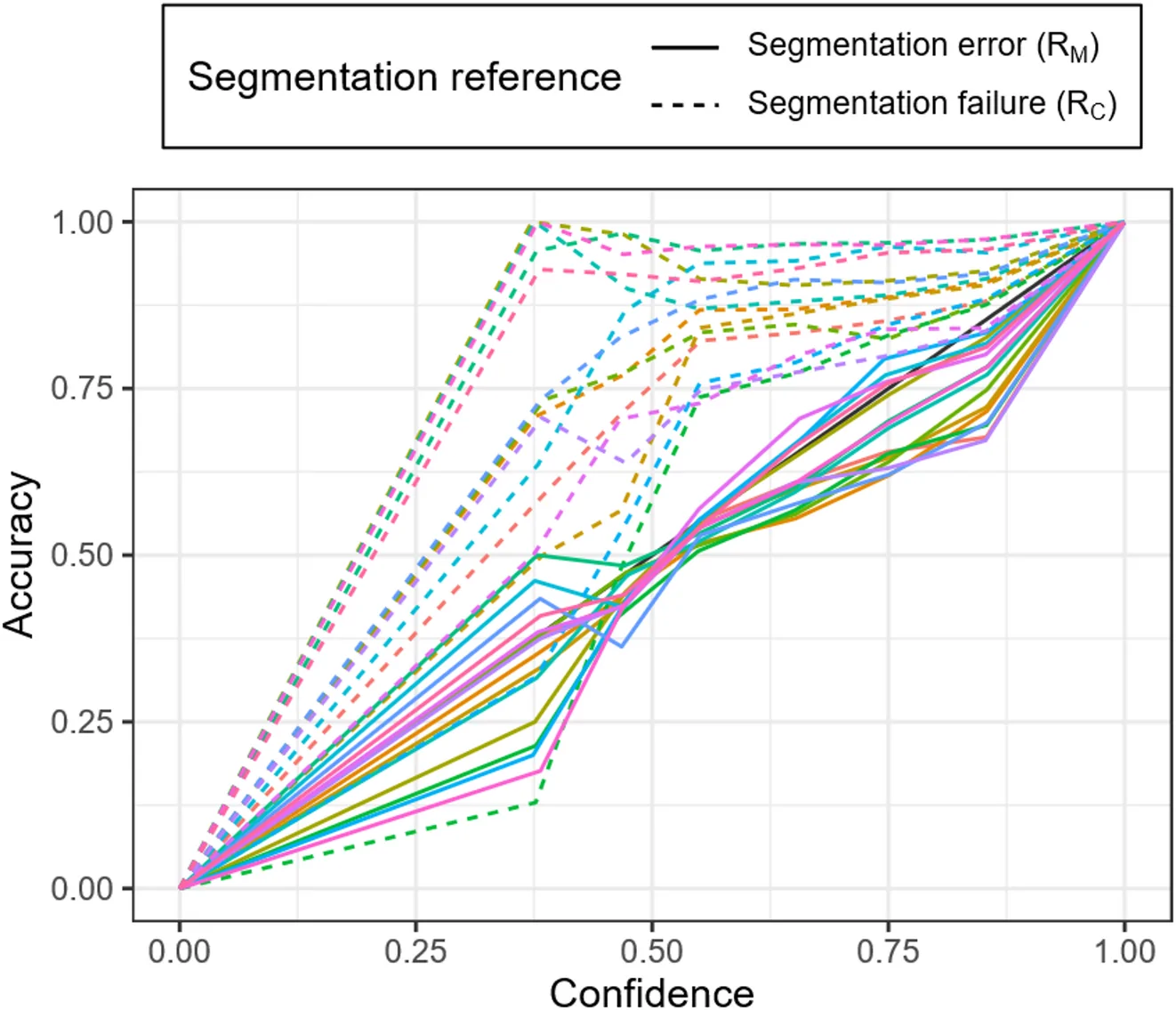
Reliability diagram per patient from the calibration set. The results are separated by considering segmentation error (R_M_) or segmentation failures (R_C_) as a reference for erroneous regions. A diagonal line in the plot indicates the optimal calibration.

On the calibration set, this configuration achieved a Dice coefficient for the cartilage of 91.6% and for the labrum of 80.3%. The mean ECE was 0.05%, the mean ACE, 3.4%, and the mean MCE, 12.7%, considering the manual segmentation as a reference. When considering segmentation failures only, the Dice coefficient was for the cartilage 96.8% and for the labrum 91.8%, the mean ECE was 0.09%, the mean ACE, 14.3%, and the mean MCE was 41%. Figure 6 shows the individual reliability diagram per patient. A low calibration error was achieved on the patient level using the segmentation error as a reference. Using the segmentation failures as a reference, the network was underconfident, resulting in a high calibration error on the patient and dataset level.

### Segmentation failure localisation

**Fig. 7:**
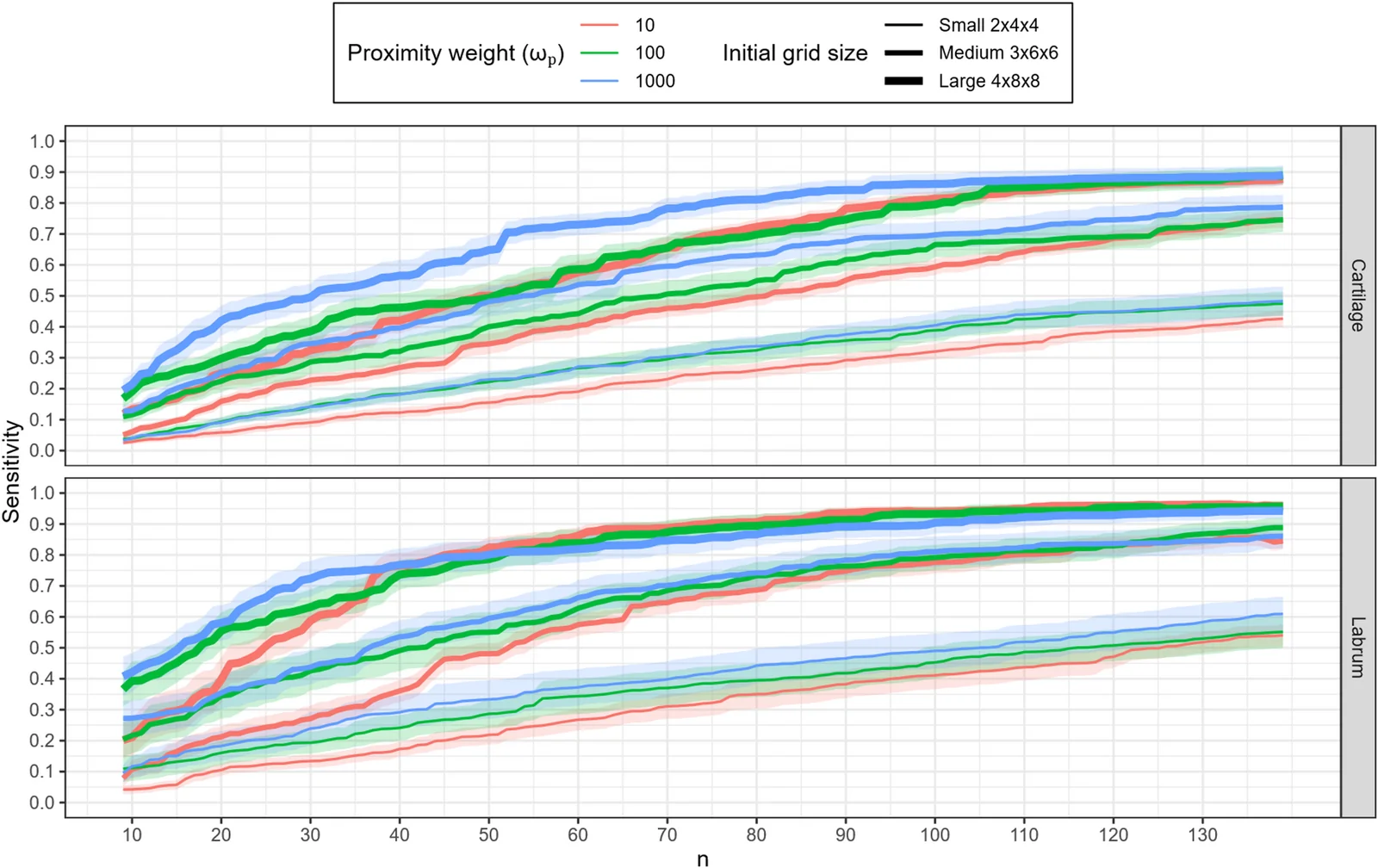
Evaluation on the calibration dataset. Sensitivity of detecting segmentation failures based on decreasing order of aggregated uncertainty in relation to the number of supervoxels (n). Cartilage and labrum were analysed separately, comparing different parameters for initial supervoxel grid size and proximity weight (ω_P_). Shaded areas indicate the standard error.

Figure 7 shows the sensitivity (average over all patients from the calibration set) of segmentation failure localisation for aggregated supervoxels in decreasing order of uncertainty. Larger supervoxel sizes improved the sensitivity of segmentation failure localisation because they covered a greater volume per patch. However, a small supervoxel size is desirable to improve the precision of manual inspection and correction by an expert. The proximity weight of 1000 achieved the greatest sensitivity for the first supervoxels. For the cartilage, this was true up to the 125th supervoxel, and for the labrum, up to the 35th supervoxel. The effect of the combining model was negligible compared to the grid size and proximity weight.

### Segmentation failure correction

#### Optimisation

**Fig. 8:**
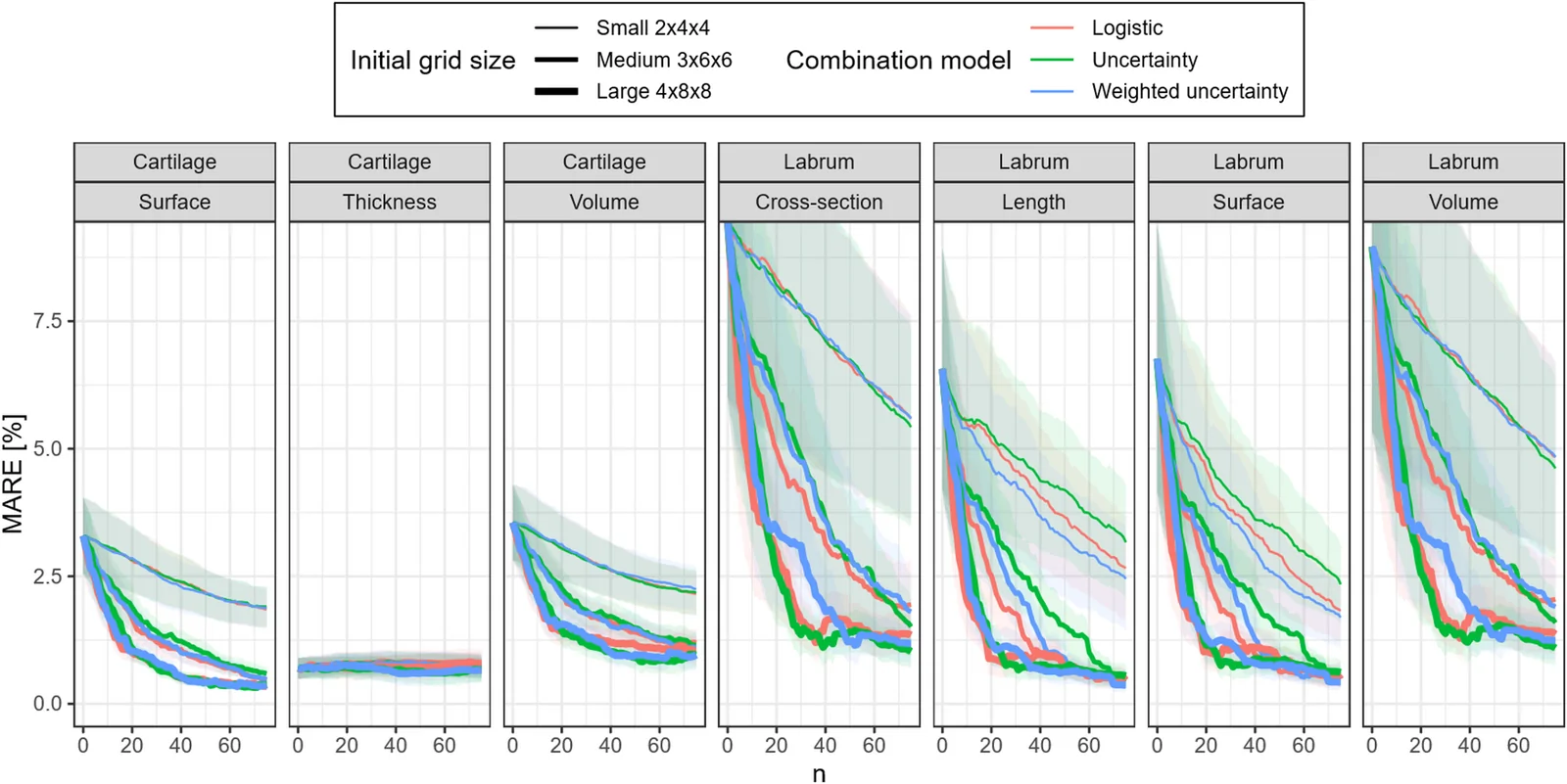
Optimization phase on the calibration dataset, selection of the combination model. Simulation of guided manual correction of localised segmentation failures to evaluate different model combinations. The predicted label was corrected within the located supervoxel. Only results with a proximity weight of 1000 are shown. The Mean Absolute Relative Error (MARE) of the clinical error was reported after each correction step n. The figure compares initial supervoxel grid sizes of 2 × 4 × 4, 3 × 6 × 6, and 4 × 8 × 8, and evaluates the three combination models: Logistic model (Logistic), uncertainty map only (Uncertainty), and uncertainty map multiplied by the mean of the metric sensitivity metric (Weighted uncertainty). Shaded areas indicate the standard error.

Figure 8 depicts the influence of the different settings for guided segmentation correction on the clinical metric accuracy for a given number of corrected supervoxels (n). Similarly to the localisation of segmentation failures, large voxel size and a proximity weight of 1000 provided optimal results (lower clinical metric error within a low number of supervoxel corrections). Multiplying the aggregated uncertainty map by the mean of the metric sensitivity maps did not improve performance compared to the aggregated uncertainty map alone. A rapid decrease of the MARE (%) occurred within the correction of approximately 25 supervoxels for the labrum and cartilage.

**Fig. 9:**
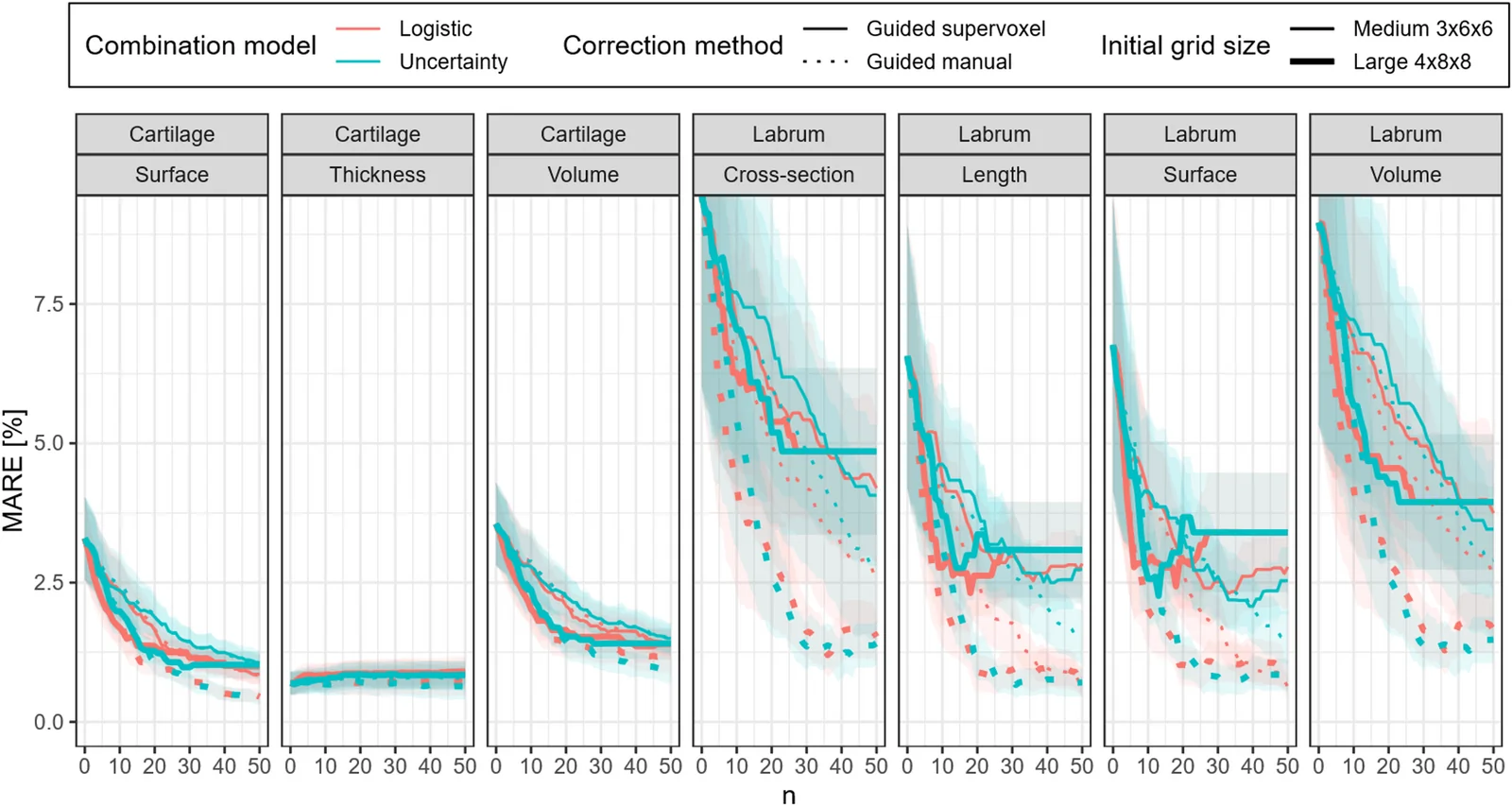
Optimization phase on the calibration dataset, comparison of correction types. Comparing the performance of guided manual versus supervoxel correction, including different initial grid sizes for the supervoxel. Results with a proximity weight of 1000, and the logistic regression and uncertainty combining method are shown. The Mean Absolute Relative Error (MARE) of the clinical error is reported after each correction step n. Logistic model (Logistic), uncertainty map (Uncertainty). Shaded areas indicate the standard error.

Figure 9 shows the performance of the guided supervoxel correction compared to guided manual correction. During the correction of the first 10 supervoxels, the performance was very similar between the guided manual and supervoxel correction. Afterwards, the guided supervoxel correction begins to lag, and the performance gap widens. For the guided manual correction, a bigger voxel size provided better results, whereas, for the guided supervoxel correction below 25 supervoxels, a 4 × 8 × 8 voxel size decreased the error faster, but after approximately 30 to 50 supervoxels, a 3 × 6 × 6 voxel size outperformed the bigger size, especially for the labrum. Use of the logistic regression model resulted in a faster error decay over correction of the first 10 supervoxels compared to the simple method of uncertainty aggregation over the supervoxels. The already low error of the cartilage thickness calculation (less than 1%) was not reduced further.

### Testing

**Fig. 10:**
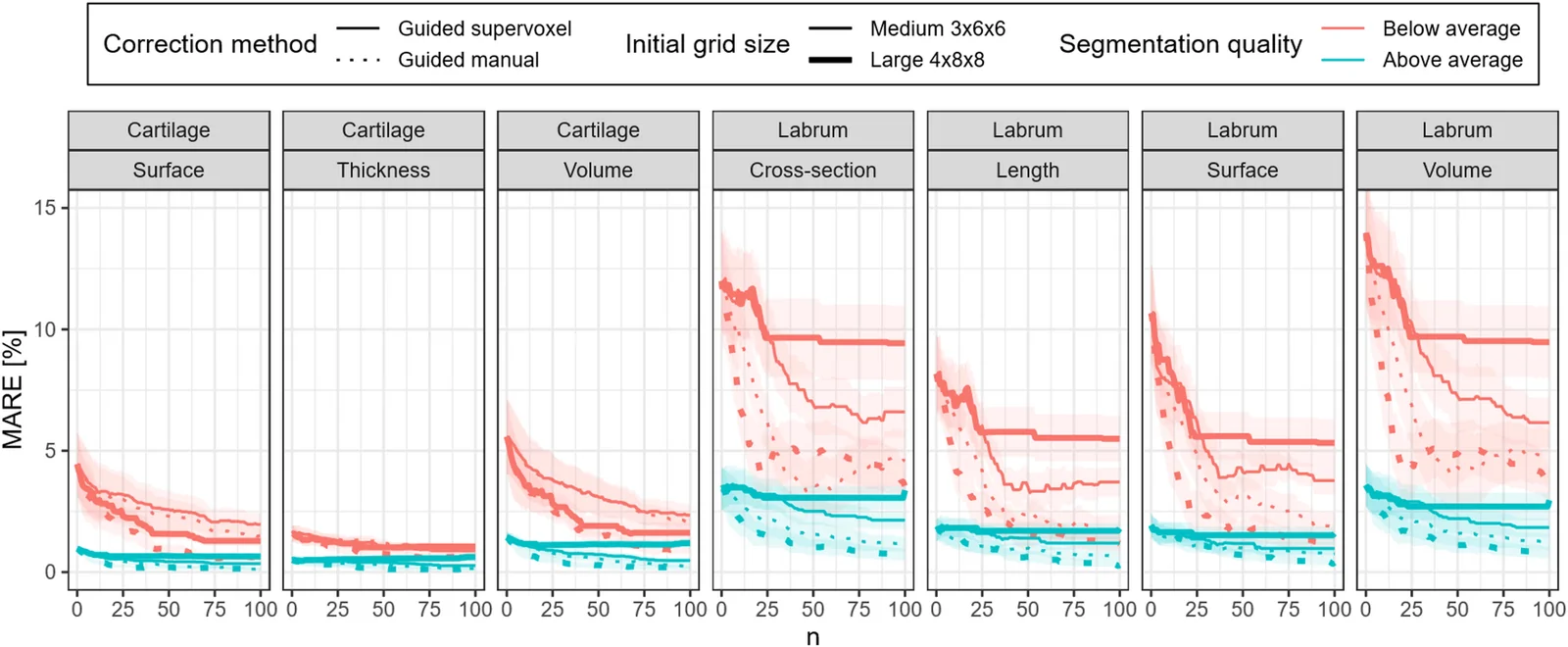
Validation phase on the independent test dataset. Comparing guided manual and guided supervoxel correction using the optimized parameters, including two initial grid sizes for the supervoxels. Settings used: Entropy of the standard nnU-Net ensemble for uncertainty estimate, proximity weight of 1000, and logistic regression model to locate segmentation failures. Results are separated into two groups: Segmentations that are above or below the average Dice coefficient to evaluate the performance for better and worse initial segmentation results. The Mean Absolute Relative Error (MARE) of the clinical error is reported after each correction step n. Shaded areas indicate the standard error.

Applying the best performing parameters (standard ensemble of the nnU-Net for segmentation, uncertainty assessment with a supervoxel proximity weight of 1000, initial grid size of 3 × 6 × 6 and 4 × 8 × 8, and use of the logistic regression combining model) to the test dataset, showed that a supervoxel size of 4 × 8 × 8 voxel is beneficial for cartilage segmentation failure localisation and guided supervoxel correction. This supervoxel size is too coarse for the labrum, and the size of 3 × 6 × 6 is better suited for a guided supervoxel correction of the labrum. On the test set, the rate of error reduction declines significantly after correction of approximately 40–50 supervoxels using the guided supervoxel correction methods (Fig. 10).

Table 1 summarises the remaining MARE for segmentations with a Dice coefficient below average, both before and after correction using 10, 25, and 50 supervoxels. After 50 supervoxels, the guided manual correction achieved an overall 3.5-fold reduction in the remaining error, while the guided supervoxel correction achieved a 2.1-fold reduction. This corresponds to a guided supervoxel error correction efficiency of 62%. Correcting the top 10 regions yielded up to 88% efficiency.

**Table 1:** The Mean Absolute Relative Error (MARE) on the test set for cartilage and labrum segmentation results below the average Dice coefficient. Comparing guided manual and guided supervoxel correction. Settings used: Entropy of the standard nnU-Net ensemble for uncertainty estimate, proximity weight of 1000, and logistic regression model to locate segmentation failures. Initial grid size for the supervoxels (GS).

Label	GS	Metric	MARE after n corrections [%]						
		Correction method	Initial	manual	supervoxel	manual	supervoxel	manual	supervoxel
		Number of corrections (n)	0	10		25		50	
Cartilage	4 × 8 × 8	Surface	4.5	2.6	2.9	1.8	2.3	0.9	1.6
	4 × 8 × 8	Thickness	1.6	1.3	1.4	1.0	1.2	0.8	1.0
	4 × 8 × 8	Volume	5.6	3.5	3.6	2.6	2.8	1.4	1.9
Labrum	3 × 6 × 6	Cross-section	12.0	9.7	11.5	6.2	9.9	3.6	6.9
	3 × 6 × 6	Length	8.2	6.4	7.3	4.2	6.0	2.2	3.5
	3 × 6 × 6	Surface	10.7	7.2	7.8	4.9	6.2	3.0	4.1
	3 × 6 × 6	Volume	14.0	10.5	12.2	6.9	10.2	4.3	7.2
Cartilage		Average	3.9	2.5	2.6	1.8	2.1	1.0	1.5
		MARE reduction factor [ ]		1.6	1.5	2.2	1.8	3.8	2.6
		Guided supervoxel correction effectiveness [%]			94		85		68
Labrum		Average	11.2	8.5	9.7	5.5	8.1	3.3	5.4
		MARE reduction factor [ ]		1.3	1.2	2.0	1.4	3.4	2.1
		Guided supervoxel correction effectiveness [%]			87		68		61
Overall		Average	8.06	5.9	6.7	3.9	5.5	2.3	3.8
		MARE reduction factor [ ]		1.4	1.2	2.1	1.5	3.5	2.1
		Guided supervoxel correction effectiveness [%]			88		71		62

## Discussion

Towards robust clinical application of deep learning-based anatomy analysis, we have proposed methods for automatic segmentation accuracy evaluation, segmentation error localisation and application-specific correction optimisation. Our methods rely on the aggregation of the CNN uncertainty over defined supervoxel regions to expedite the correction process, eliminating the need for voxel-wise correction. Our empirical results demonstrate that the primary parameters driving segmentation failure localisation are the supervoxel grid size and proximity weight. Furthermore, the integration of metric sensitivity maps acts as a helpful secondary feature to focus correction on important areas. While the overall effect of the combining model is limited compared to uncertainty aggregation alone, it results in a faster error decay over the correction of the first 10 supervoxels, guiding the user’s attention to areas where the impact of the correction is the highest initially.

We found that the standard ensemble of the nnU-Net framework generates confidence levels that align with their respective segmentation errors for cartilage and labrum segmentation. Within the nnU-Net framework, this comes with no additional computational costs. Compared to the findings of Jungo et al.^9^, we observed good calibration results per patient, in addition to good accuracy over the entire dataset. However, a distinct behaviour was observed between general segmentation errors (R_M_) and gross segmentation failures (R_C_). The network is well-calibrated for segmentation errors, as it effectively captures the inter-rater variability inherent in the manual training labels; the uncertainty correctly highlights ambiguous tissue borders where experts might disagree. In contrast, when evaluated against segmentation failures, where we removed minor boundary variations to focus on larger, clinically relevant errors, the network demonstrated larger miscalibration and under-confidence. This suggests the network lacks the broader anatomical context to distinguish real structural segmentation failures from acceptable boundary variability. Despite this voxel-wise miscalibration regarding structural segmentation failures, our results indicate that the system remains clinically reliable through the aggregation process. By aggregating voxel-wise uncertainties over feature-derived supervoxels, the signal of potential errors is amplified. While the absolute probability values for failures may be under-confident, the relative ranking of the supervoxels remains robust. This allows the system to correctly prioritise regions that are most likely to negatively impact the clinical diagnosis, ensuring that the user’s attention is guided to the most critical areas first.

Compared to a slice-by-slice verification, the 3D supervoxels employed in our method allow for a fast correction of a similar area in 3D. We achieved an approximate sensitivity of 0.8 in the detection of segmentation errors, within the first 50 to 100 supervoxels per MRI volume, depending on grid size and tissue type. While more than 50 corrections might seem substantial, it represents a significant improvement over the state of the art. Sander et al.^7^ reported over 100 false positive 2D patches to reach similar accuracies, resulting in significantly more regions to correct. Given that the in-plane image resolution and patch size of the datasets analysed in this work were comparable to those analysed by Sander et al., the advantages of using 3D regions over 2D patches are evident.

In our study, after correcting 50 supervoxels per MRI in the test set, the guided manual correction resulted in a 3.5-fold reduction in MARE, while the guided supervoxel correction method resulted in a 2.1-fold reduction. This indicates that approximately 62% of the error reduction can be achieved through the simple and efficient correction at the supervoxel level. For only 10 corrections, this guided supervoxel correction efficiency increased to 88%. The definition of the supervoxel size represents a critical trade-off: regions must be as large as possible to reduce workload, yet small enough for precise localization. This balance is structure-dependent. Cartilage, which comparatively smooth geometry and clearer boundaries, allowed more robust supervoxel-level correction. In contrast, the labrum is a curved, tubular structure and often shows lower contrast relative to the acetabulum and capsule. The rapid decay of the MARE for the labrum metrics suggests that our method may be particularly well-suited to identifying segmentation failures when geometry-sensitive metrics are involved, such as the surrogate measure of the labrum’s angular coverage (α) on the clockface. Building on this, we anticipate that the logistic model will hold potentially greater utility in future applications involving highly localized or geometry-sensitive metrics.

We acknowledge that for tubular structures like the labrum, where connectivity is a primary concern, evaluating single supervoxels in isolation has limitations. Segmentation failures in these structures are often spatially correlated. In our study, we restricted our localisation and correction methods to supervoxels directly in contact with the segmented structure In future work, we suggest an iterative evaluation of the supervoxels, dynamically updating the metric sensitivity as corrections are made, to better address these specific geometric constraints. This approach would enable the algorithm to address larger error regions or detached areas and account for connectivity constraints. Additionally, we optimised the logistic model to minimise segmentation failures rather than focusing on a specific metric. Due to the simplicity of the model, users could adjust the corresponding weights of the chosen clinical metric, allowing them to optimise the correction process for specific clinical needs.

In this work, we simulated ideal corrections scenarios for both the guided manual and guided supervoxel correction process, using the ground-truth segmentation to ensure perfect corrections. While this approach allows for a direct comparison of the methods, it does not account for the impact of imperfect corrections, cognitive load, context-switching fatigue between spatially separated regions, or the time required for a user to perform them. In a future study, we will implement our findings in user-friendly software to evaluate our method’s effectiveness in a real-world clinical workflow. Although we did not measure the actual time savings or direct clinical benefit in this study, we assume that guiding the user to a limited number of 3D regions could potentially reduce the overall correction burden compared to exhaustive slice-by-slice inspections. In a practical application, user interaction with supervoxels could be as simple as mouse clicks or scribbles or it could serve as a guided prioritization tool, potentially reducing user interaction significantly and enabling larger areas to be corrected quickly. Our approach deliberately avoids more complex methods, such as machine learning models, to ensure the application remains straightforward, transparent, and intuitive for the end user. This design also allows flexibility in adjusting the weights in the logistic regression function, tailoring the methods to specific clinical metrics.

The goal of this work was to investigate methods that would improve the robustness of automatic deep learning-based clinical measures by empowering the user with additional insights, such as network uncertainty and estimated sensitivity metrics. The methods have the potential to streamline and accelerate the correction process by combining machine efficiency with human expertise and by optimising correction for metric-specific accuracy requirements. Our study demonstrates that by aggregating uncertainties across feature-derived regions, we can achieve high sensitivity in identifying segmentation errors. Furthermore, the guided supervoxel correction method shows significant promise in easing the correction process and reducing the end user’s workload.

Our current evaluation focuses on automatic hip cartilage and labrum analysis. Adapting the approach to other anatomical structures will at least require a new calibration set to optimize the supervoxel parameters and fine-tune the logistic model for the new task. Generation of sensitivity maps efficiently also require quick calculations of the clinical metric, as this process must be repeated multiple times to evaluate the impact of supervoxel-defined regions. For instance, a deep learning-based landmark detection algorithm that uses segmentation as input would be too slow for this purpose. Instead, we suggest that after initial detection of the landmark, the dependent clinical metric could be re-evaluated based on the shift of the landmark caused by adjustments in the segmentation results.

## Conclusion

This work presents a proof-of-concept for the clinical application of deep learning for anatomy analysis by introducing methods for automatic segmentation evaluation, error localisation, and targeted correction in hip MRI. Leveraging CNN uncertainty aggregated over 3D supervoxels, our approach has the potential to streamline corrections, focusing on areas where improvements have the most clinical impact. While combining metric sensitivity maps and aggregated uncertainty to risk scores provides a secondary benefit in guiding initial corrections, with potentially greater utility for highly localized clinical metrics, the core strength of this method lies in the 3D feature-derived uncertainty aggregation to potentially minimize manual intervention.

We demonstrated that the nnU-Net can produce reliable confidence levels and achieved efficient error detection and correction using 3D supervoxels, with high sensitivity and significant error reduction for segmentation of the hip cartilage and labrum. Compared to traditional 2D patch methods, this 3D approach substantially reduces the number of targeted regions requiring manual review and has the potential to allow for faster corrections in future clinical workflows.

## Data Availability

The data that support the findings of this study are available on request from N.G. The data is not publicly available due to the contained information that could compromise the privacy of the research participants.
